# Fulminant Vasculitis Complicated by ST-Elevation Myocardial Infarction and Stroke

**DOI:** 10.1155/2021/8365283

**Published:** 2021-08-16

**Authors:** Vy A. Pham, Christopher P. Kovach, Karl Gordon Patti, Evan S. Manning, Stephen J. Slade, Rebecca Pollard, Elena Weinstein, Jennifer Simpson, James E. Carter

**Affiliations:** ^1^Department of Medicine, University of Colorado, 12631 E. 17th Ave, Room 8601, Aurora, CO 80045, USA; ^2^Division of Cardiology, Department of Medicine, University of Colorado, 12631 E. 17th Ave, B130, Aurora, CO 80045, USA; ^3^Division of Rheumatology, Department of Medicine, University of Colorado, 1775 Aurora Court, B115, Aurora, CO 80045, USA; ^4^Division of Neurology, Department of Medicine, University of Colorado, 12700 E. 19th Ave, Room 5018, Aurora, CO 80045, USA

## Abstract

Acute coronary syndrome is a rare complication of vasculitis. We present a case of fulminant medium-vessel vasculitis, most likely PAN, complicated by STEMI and stroke, that was successfully treated with percutaneous revascularization, high-quality stroke care, and immunosuppression. This case highlights the importance of prompt diagnosis and treatment of vasculitis and the recognition of coronary and cerebral ischemia as potentially serious complications.

## 1. Introduction

Systemic vasculitides are rarely associated with acute myocardial infarction or stroke. Prompt diagnosis and treatment is critical given the increased morbidity and mortality of systemic vasculitis with cardiovascular involvement [1].

## 2. Case Report

**Figure 1:**
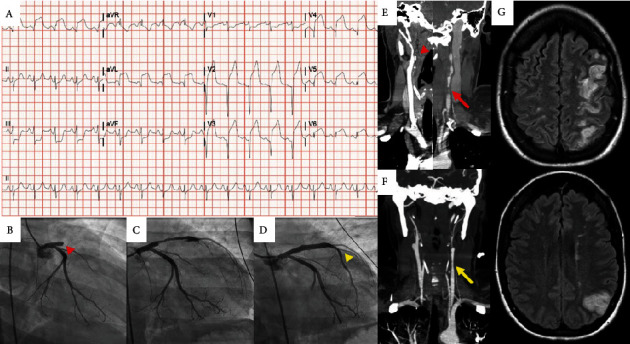
Cardiovascular and neurovascular imaging. Electrocardiogram (A) demonstrating anterolateral STEMI. Coronary angiography demonstrated complete occlusion of the LAD (B; red arrowhead) with partial reconstitution of flow following wiring (C) and restoration of normal flow following stent deployment (D; yellow arrowhead). CT angiography (E) demonstrated complete occlusion of the right ICA (red arrowhead) with luminal narrowing and irregularity of the left ICA consistent with dissection and associated thrombus (red arrow). Subsequent CT angiography showed improvement of the left ICA (F, yellow arrow). MRI demonstrated left frontal and parietal lobar multifocal infarction (G).

A 30-year-old woman with a one-year history of recurring painful lower extremity skin lesions presented with acute headache, neck pain, and right arm weakness with paresthesia. She was tachycardic but afebrile and normotensive. CT angiography of the head and neck revealed high-grade stenosis of the internal carotids. Subsequently, the patient reported acute chest pain and dyspnea. ECG revealed sinus tachycardia, anterior ST elevation, and reciprocal inferior depression (Figure 1). Labs revealed elevated troponin, serum lactate, and white blood cell count (Table 1). She was given aspirin and intravenous bivalirudin and underwent emergent cardiac catheterization that revealed an acute occlusive thrombus of the proximal left anterior descending (LAD) artery and estimated Fick cardiac index 1.9 L/min/m^2^ (Figure 1, Table 1). Aspiration thrombectomy was performed, and a 4.5 × 30 mm drug-eluting stent was successfully deployed in the proximal LAD. The patient was started on a norepinephrine infusion and transferred to our institution with concern for fulminant medium-vessel vasculitis complicated by ST-elevation myocardial infarction (STEMI), cardiogenic shock, and stroke.

Upon arrival to our hospital, exam demonstrated expressive aphasia, left upper quadrantanopia, and right-sided hemiparesis. Bilateral upper extremity pulses were normal, femoral pulses were reduced, and dorsalis pedis and posterior tibial pulses were absent. A pulmonary arterial catheter was placed (Table 1). Neurology was consulted and urgent head and neck CT angiography revealed high-grade occlusions of the bilateral internal carotid arteries and stigmata of left middle cerebral artery infarct (Figure 1). Brain MRI confirmed evolving left frontal and parietal lobe infarcts (Figure 1). Echocardiogram demonstrated severe left ventricular systolic dysfunction with regional wall motion abnormalities: anterior, septal, and apical. CT imaging demonstrated abnormal wall thickening of the thoracic aorta, common iliac arteries, and renal arteries, as well as occlusion of the inferior mesenteric and infratibial arteries. Rheumatology was consulted and a broad serologic evaluation was initiated (Table 1).

### 2.1. Management

The patient was treated with IV corticosteroids and cyclophosphamide. Shock physiology, likely secondary to myocardial stunning, rapidly resolved. Systemic anticoagulation was discontinued after 48 hours following the determination that arterial occlusions were attributable to vasculitis rather than thrombophilia. Dual antiplatelet therapy with clopidogrel and aspirin was continued.

### 2.2. Follow-Up

On hospital day 10, the patient was transferred to acute rehabilitation for poststroke care. She was prescribed prednisone, cyclophosphamide, maintenance dual antiplatelet therapy, and a heart failure regimen. Rheumatology recommended outpatient adenosine deaminase 2 (*ADA2*) genetic testing. Three months after discharge, she was doing well on maintenance immunosuppression and following with cardiology for chronic heart failure.

## 3. Discussion

Cardiac involvement in vasculitis is not uncommon and can include cardiomyopathy, pericarditis, valvular disease, arrythmia, aortic dissection, and coronary complications such as aneurysm, stenosis, thrombosis, or rupture [1]. However, STEMI is an uncommon initial presentation of vasculitis. This report highlights the diagnosis and management of fulminant medium-vessel vasculitis, with features of polyarteritis nodosa (PAN) or adenosine deaminase 2 deficiency (DADA2), presenting as STEMI and acute stroke.

PAN is a systemic medium-vessel necrotizing vasculitis that most frequently involves the skin, kidneys, and peripheral nervous system and is diagnosed by a combination of clinical, angiographic, and pathologic findings [2]. Cardiovascular involvement occurs in only 5-22% of patients and carries a two to threefold higher mortality compared to those without coronary involvement [3]. Coronary involvement in PAN is angiographically characterized by alternating areas of stenosis or occlusion with aneurysm in a “beads on a string” pattern [1, 2]. Treatment involves aggressive immunosuppression, typically with corticosteroids and cyclophosphamide [2]. DADA2 is an autoinflammatory disorder first described in 2014 that clinically resembles PAN and is characterized by vasculitis, dysregulated immune function, and hematologic abnormalities [4, 5]. Pathophysiologically, lack of ADA2 leads to proinflammatory endothelial dysfunction, predisposing patients to systemic vasculitis and recurrent strokes, often beginning in childhood [6]. Significant clinical overlap and lack of commercial enzyme testing makes differentiating ADA2 deficiency from PAN challenging, though genetic testing for *CECR1* mutations can aid in diagnosis. While there is no clearly preferred immunosuppressive therapy for DADA2, tumor necrosis factor inhibitors may be particularly effective [5].

Takayasu's arteritis (TA) is a large-vessel vasculitis primarily involving the aorta and its major branches that usually affects young women with preexisting cardiac abnormalities [1]. Coronary artery stenosis and occlusions usually occur at the ostia because of the extension of inflammation-induced intimal proliferation and subsequent fibrotic retraction from the ascending aorta [7]. Treatment involves high-dose immunosuppression. TA was felt to be less likely in this patient given the predominantly medium-vessel involvement encompassing multiple vascular territories beyond the aorta.

The patient's clinical presentation, imaging, and laboratory findings were inconsistent with alternative diagnoses such as Behcet's disease, giant cell arteritis, or other systemic rheumatologic conditions.

## 4. Conclusions

Acute coronary syndrome is a rare complication of vasculitis. Our patient presented with fulminant medium-vessel vasculitis, most likely PAN, complicated by STEMI and stroke, and was successfully treated with percutaneous revascularization, high-quality stroke care, and immunosuppression. This case highlights the importance of prompt diagnosis and treatment of vasculitis and the recognition of coronary and cerebral ischemia as potentially serious complications.

## Figures and Tables

**Table 1:** Clinical, laboratory, and echocardiographic data.

	Day 1	Day 2	Day 4
Vitals			
Temperature, °C	35.6	36.2	36.3
Heart rate, beats/min	150	143	98
Blood pressure, mmHg	120/105	123/87	113/73
Respiratory rate, breaths/min	25	30	22
SpO_2_, %	100	98	99
Body mass index, kg/m^2^	32	32	32

Laboratories			
Troponin I, ng/L	7.0	—	—
Hs-troponin, ng/L	—	206,452	174,327
NT-proBNP, ng/mL	—	1,059	—
White blood cells, 10^9^/L	53	25	16
Hemoglobin, g/dL	16	13	10
Platelets, 10^9^/L	460	364	254
Creatinine, mg/dL	0.60	0.80	0.50
Aspartate transaminase, IU/L	1,478	533	113
Alanine aminotransferase, IU/L	158	120	53
International normalized ratio	5.1	2.0	1.1
Lactate, mmol/L	6.2	3.6	1.8
Erythrocyte sedimentation rate, mm/hr	N/A	58	—
C-reactive protein, mg/dL	2.9	125	—
Hemoglobin A1C, %	5.5	5.4	—
Total cholesterol, mg/dL	193	137	—
Triglycerides, mg/dL	224	146	—
HDL cholesterol, mg/dL	44	33	—
LDL cholesterol, mg/dL	104	79	—

Echocardiogram			
LVEDV, mL	115		174
LVESV, mL	89		110
LVEF, %	23		37
Left atrial volume, mL/m^2^	15.5		15.3
RVSP, mmHg	—		44.6
Valvular heart disease	None		None

Cardiac hemodynamics			
RA pressure, mmHg	3	5	5
PA mean pressure, mmHg	26	25	27
PCW pressure, mmHg	—	15	—
Cardiac output, L/min	4.1	3.8	5.7
Cardiac index, L/min/m^2^	1.9	1.7	2.6

Infectious studies			
HIV			Negative
Hepatitis B DNA PCR			Negative
Hepatitis C			Negative
Blood cultures			Negative

Rheumatologic studies			
ANA titer			1 : 320, speckled
Anti-ds-DNAb			Negative
Anti-Smith			Negative
Anti-RNP			Negative
Anti-SSA			Negative
Anti-SSB			Negative
ANCA IgG			Negative
Antimyeloperoxidase			Negative
Antiproteinase 3			Negative
C3			Normal
C4			Normal
Lupus anticoagulant			Negative
Beta-2 glycoprotein-1 IgG/IgM			Negative

Abbreviations: ANA: antinuclear antibody; ANCA: antineutrophil cytoplasmic antibodies; ds-DNA: double stranded DNA; HDL: high-density lipoprotein; HIV: human immunodeficiency virus; Hs: high sensitivity; LDL: low-density lipoprotein; LVEDD: left ventricular end diastolic diameter; LVEDS: left ventricular end systolic dimension; LVEF: left ventricular ejection fraction; MR: mitral regurgitation; RNP: ribonucleoprotein; RVSP: right ventricular systolic pressure; SSA/SSB: Sjogren's syndrome-related antigen A/B.

## Data Availability

Data supporting the conclusion may be made available via communication with the corresponding author.

## Additional Points

*Learning Objectives*. (1) Recognize vasculitis as a rare etiology of STEMI. (2) Recognize vasculitis as an uncommon etiology of stroke. (3) Emphasize necessity of early recognition of vasculitis and prompt immunosuppression.
